# Increased type II collagen cleavage by cathepsin K and collagenase activities with aging and osteoarthritis in human articular cartilage

**DOI:** 10.1186/ar3839

**Published:** 2012-05-14

**Authors:** Valeria M Dejica, John S Mort, Sheila Laverty, John Antoniou, David J Zukor, Michael Tanzer, A Robin Poole

**Affiliations:** 1 Genetics Unit, Shriners Hospitals for Children, 1529 Cedar Avenue, Montreal, QC H3G 1A6, Canada; 2 Department of Surgery, McGill University, 1650 Cedar Avenue, Montreal, QC H3G 1A4, Canada; 3 Département de sciences cliniques, Faculté de Médecine Vétérinaire, Université de Montréal, CP 5000, St. Hyacinthe, QC J2S 7C6, Canada; 4 Lady Davis Institute for Medical Research, SMBD-Jewish General Hospital, 3755 Chemin de la Cote Ste-Catherine, Montreal, QC H3T 1E2, Canada; 5 Division of Orthopaedic Surgery, Montreal General Hospital, 1650 Cedar Avenue, Montreal, QC H3G 1A4, Canada

## Abstract

**Introduction:**

The intra-helical cleavage of type II collagen by proteases, including collagenases and cathepsin K, is increased with aging and osteoarthritis (OA) in cartilage as determined by immunochemical assays. The distinct sites of collagen cleavage generated by collagenases and cathepsin K in healthy and OA human femoral condylar cartilages were identified and compared.

**Methods:**

Fixed frozen cartilage sections were examined immunohistochemically, using antibodies that react with the collagenase-generated cleavage neoepitopes, C2C and C1,2C, and the primary cleavage neoepitope (C2K) generated in type II collagen by the action of cathepsin K and possibly by other proteases, but not by any collagenases studied to date.

**Results:**

In most cases, the staining patterns for collagen cleavage were similar for all three epitopes: weak to moderate mainly pericellular staining in non-OA cartilage from younger individuals and stronger, more widespread staining in aging and OA cartilages that often extended from the superficial to the mid/deep zone of the tissue. In very degenerate OA specimens, with significant disruption of the articular surface, staining was distributed throughout most of the cartilage matrix.

**Conclusions:**

Cleavage of collagen by proteases usually arises pericellularly around chondrocytes at and near the articular surface, subsequently becoming more intense and extending progressively deeper into the cartilage with aging and OA. The close correspondence between the distributions of these products suggests that both collagenases and cathepsin K, and other proteases that may generate this distinct cathepsin K cleavage site, are usually active in the same sites in the degradation of type II collagen.

## Introduction

Type II collagen (Col II), the major structural component of the articular cartilage, is composed of three identical α chains that form collagen fibrils. The mechanical properties and tensile strength of cartilage depend to a large degree on the integrity of the mesh-like endoskeleton formed by bundles of collagen fibrils with different orientations and diameters in each of the four zones of articular cartilage [1]. Under non-inflammatory conditions, collagen turnover is mediated by the only cell type found in articular cartilage, the chondrocyte. Increased catabolic responses in osteoarthritis (OA) mediated by chondrocytes involve enhanced cleavage of type II collagen by collagenases [2].

Until recently, collagenases were the only connective tissue proteases known to initiate the intra-helical cleavage and denaturation of type II collagen but there is emerging evidence that collagen degradation in OA [3-6] and inflammatory arthritis [7] may also involve action of cathepsin K, a member of the papain superfamily of cysteine proteases, which is capable of cleaving triple helical fibrillar collagens at multiple sites, [8,9] other than the collagenase cleavage site [4].

**Figure 1:**
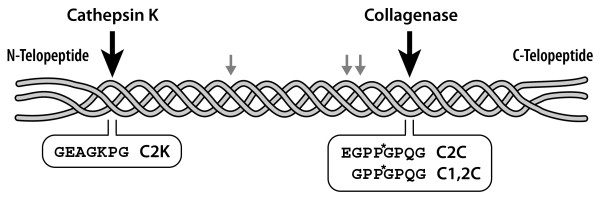
**Peptide sequences used for the preparation of collagen cleavage antibodies**. The cleavage positions of cathepsin K and collagenase in the triple helical domain of type II collagen are indicated. Small grey arrows designate the minor collagenase cleavage positions recognized by the C1,2C antibody following reaction at the primary site. The sequences of the collagen molecule forming the C-terminus of the fragments generated on cleavage represent the C2K, C2C and C1,2C epitopes. P* denotes 4-hydroxyproline.

Damage to articular cartilage results in the loss of the tensile properties, which is determined by the collagen fibrillar network [10]. Cartilage swelling and deformation associated with cartilage collagen degradation is one of the hallmarks of early OA [11]. Articular chondrocytes express collagenases (MMP-1, 8, 13 and 14) that belong to the matrix metalloproteinase (MMP) family and the cysteine protease cathepsin K, both of which are capable of cleaving the triple helical region of type II collagen at specific and distinct sites [8,12]. Collagenases are known to cleave the collagen triple helical monomers towards the C-terminal end, resulting in the generation of three-quarter and one-quarter length fragments. Cathepsin K, on the other hand, is known to cleave not only Col II extrahelical regions (telopeptides), resulting in fibril depolymerization, but also the triple-helical region, for example, at a site located 58 residues from the N-terminus of the triple helix [8] (Figure 1). This cleavage pattern is considered unique among other proteases and it does not depend on previous destabilization of the triple helix. Collagenases, such as MMP-13, do not generate this cleavage [4].

Antibodies raised against the C-terminal neoepitopes generated in the triple-helical region of the collagen fibril by cathepsin K and by collagenases were used in the development of immunochemical assays that quantitate cleavage at these sites. The levels of both of these neoepitopes in extracts of human articular cartilage were shown to be significantly increased in aging and an even greater increase was seen in OA. Moreover, generation of the cathepsin K neoepitope in OA cartilage was arrested in culture by a specific cathepsin K inhibitor [4]. Together these observations suggest that cathepsin K is involved in the generation of this neoepitope and that it can be involved in collagen cleavage in OA cartilage.

The purpose of the present study was to identify and compare, using immunohistochemistry and specific anti-neoepitope antibodies, the locations of these two distinct intrahelical cleavage sites in Col II that are generated either by collagenases or by cathepsin K in both healthy aging and OA cartilages.

## Materials and methods

### Tissue

Human femoral condylar cartilages that appeared macroscopically normal were obtained at autopsy from 12 individuals of both sexes in the age range of 19 to 69 years. Full depth cartilage (0.5 to 1.0 cm^2 ^surface area) was removed with a scalpel within 15 h of death. OA cartilage was obtained at total knee arthroplasty [2] from seven patients (55 to 75 years) with OA diagnosed in accordance with the criteria of the American College of Rheumatology [13]. Samples were stored at -20°C prior to use. Ethical approval from the McGill University Institutional Research Board was granted for these studies and informed consent was obtained from the patients or next of kin for all samples used.

### Antibodies

The antibodies that were used to assess the degradation of type II collagen by collagenases have been described previously (Figure 1). They include a mouse monoclonal antibody against the collagenase-generated primary cleavage neoepitope (C2C, previously termed COL2 3/4C(long)) [14] and rabbit polyclonal antibodies against the primary collagenase-generated C1,2C epitope (previously termed COL2-3/4C(short)) [2], which also has the potential to recognize this neoepitope present at three other sites in the α chains of type II collagen once the triple helix has been cleaved. A rabbit polyclonal antiserum raised against the C-terminal neoepitope (C2K) of the short α chain fragment generated by cathepsin K (but not by collagenases) in type II collagen [4] was used to identify the location of this cleavage site. A BLAST search has revealed that this antiserum could also recognize the same neoepitope sequence in the type I collagen α1 chain although there is no direct evidence for cathepsin K cleavage of type 1 collagen at this site. While type II is the predominant collagen in articular cartilage, the presence of minor amounts of type I has been reported based on immunohistochemical studies [15]. However, no evidence for the mRNA for type I chains was found in osteoarthritic cartilage [16].

### Immunohistochemistry

The methods used for immunolocalization of extracellular matrix in articular cartilages were similar to those described previously [17,18]. Briefly, the tissue samples were thawed, mounted and refrozen in OCT embedding media, then cryosectioned (6 μm thick sections) using a Tissue-Tek II cryostat. Sections were either refrozen and stored at -20°C or used immediately. The variable nature of the clinical material available limited our ability to obtain serial sections. For comparative purposes, an effort was made to use sections from as close as possible within the tissue sample, ideally two or three sections. Fixation of the sections and subsequent procedures were performed at room temperature. Samples were fixed for 5 minutes in 4% formaldehyde prepared fresh from paraformaldehyde hydrolyzed in PBS, followed by 3 × 10-minute washes in PBS. The permeability of the cartilage matrix was enhanced by treating with chondroitinase ABC (ICN/Flow Laboratories, Mississauga, ON, Canada) at 0.0125 U/50 μl per section in 0.1 M Tris-acetate buffer, pH 7.6, for 90 minutes at 37°C to remove glycosaminoglycans. This was followed by 1 h incubation in 0.2 M EDTA in 50 mM Tris, pH 7.6 for removal of calcified deposits, which would otherwise non-specifically bind immunoglobulins. Endogenous peroxidase activity was blocked by incubation of the sections with freshly prepared 0.5% v/v H_2_O_2 _in absolute methanol for 10 minutes followed by washing in PBS for 15 minutes. Unreactive aldehyde groups were blocked with 5% normal pig serum in PBS for 30 minutes. For the detection of the cathepsin K-induced neoepitope, the sections were incubated for 30 minutes at room temperature with 50 μl/section of rabbit antiserum R771 diluted 1:200 in PBS, 1% w/v BSA [4]. Two types of controls were prepared, one by using non-immune rabbit serum and the other by preincubating the polyclonal antiserum with the 100 μg/ml of the immunizing peptide, as described [17]. The secondary antibody consisted of biotinylated pig anti-rabbit F(ab') (Dako Cedarlane, Hornby, ON, Canada), diluted 1:800 in PBS, 1% w/v BSA. After 30 minutes incubation with the secondary antibody, the sections were washed 3 × 10 minutes in PBS and further incubated for 20 minutes with the indicator, a streptavidin-horseradish peroxidase conjugate (Amersham Biosciences, Baie d'Urfe, QC, Canada), diluted 1:300.

The same technique was used for the detection of collagenase-induced cleavage of Col II by using diluted rabbit antiserum (1:200) raised against the C1,2C neoepitope and diluted mouse ascitic fluid (1:200) raised against the C2C neoepitope. The secondary antibody for the C2C neoepitope was biotinylated rabbit anti-mouse F(ab'), diluted 1:600. The peroxidase reaction was performed with copper-H_2_O_2_/silver intensification of the nickel-diaminobenzidine end-product of the peroxidase reaction as previously described [19]. After washing, the tissue sections were mounted with VectaMount mounting medium (Vector Laboratories, Burlington, ON, Canada) and examined with light microscopy.

## Results

Full-depth articular cartilage sections were examined from 12 non-arthritic individuals of age 19 to 69 years and from 7 OA patients of age 55 to 75 years. Collagenase generated cleavage products were detected using the polyclonal antibody to the C1,2C epitope (Figure 1), which is specific for the C-terminus of the initial proteolytic product and a monoclonal antibody to the C2C epitope. We used both reagents since they recognize neoepitopes of varying length and their combined use has never been reported previously. The monoclonal antibody is specific for type II collagen since it only recognizes the C2C neoepitope that resides at the C- terminus of the TC^A ^fragment produced by cleavage of type II collagen α_1 _monomer by collagenases [14]; whereas, the polyclonal antibody recognizes the shorter neoepitope, which results from secondary cleavage generating the C1,2C epitope and is found in both type II and type I collagen [2]. The cathepsin K derived Col II neoepitope was localized using the C2K antibody.

**Figure 2:**
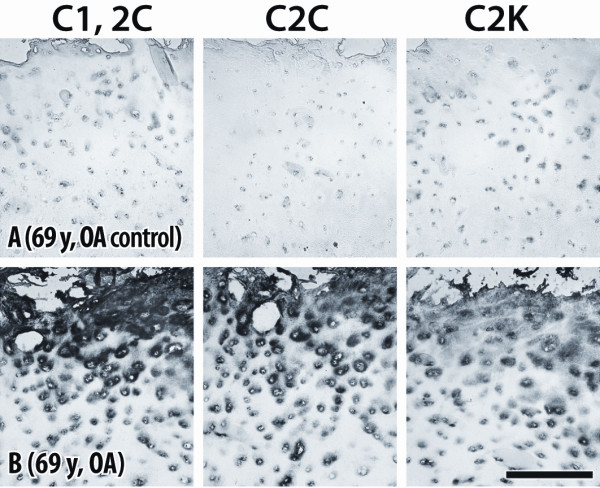
**Demonstration of antibody specificity**. High magnification views of articular cartilage from a 69-year-old OA patient, showing cleavage of type II collagen by collagenases (C1,2C and C2C) and cathepsin K (C2K). Control **(A) **was prepared by prior absorption of the antisera with the immunizing peptides. Immune staining **(B) **using unabsorbed antibodies. Bar = 200 μm.

We describe below the extracellular staining which is of particular interest and was found to be specific, based on control studies involving immunogen preabsorption of the polyclonal and monoclonal antibodies. In all cases, very weak extracellular staining was observed when the sections were treated with non-immune rabbit sera or with antisera/ascitic fluid preincubated with the peptides against which the antibodies were raised. For example, treatment of OA cartilage with pre-absorbed antibodies (Figure 2), resulted in very weak and diffuse staining compared to the very intense staining obtained when cartilage sections were incubated with the unabsorbed antibodies, demonstrating the specificity of extracellular staining.

**Figure 3:**
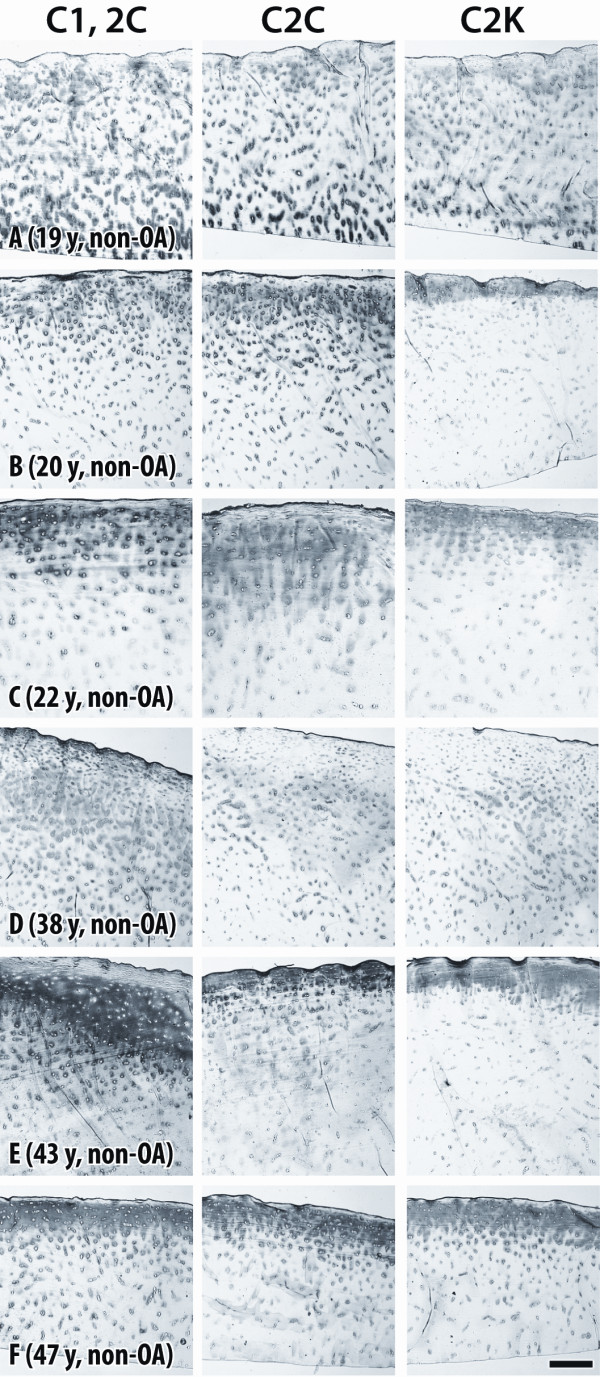
**Immunostaining of cartilage from young normal individuals**. Sections of human femoral condylar cartilage from six non-OA individuals (ages 19 to 47 years), showing detection of neoepitopes generated by collagenases (C1,2C and C2C) and cathepsin K (C2K) in type II collagen. Specimens are positioned so that the articular surface is at the top of each figure. Bar = 200 μm.

**Figure 4:**
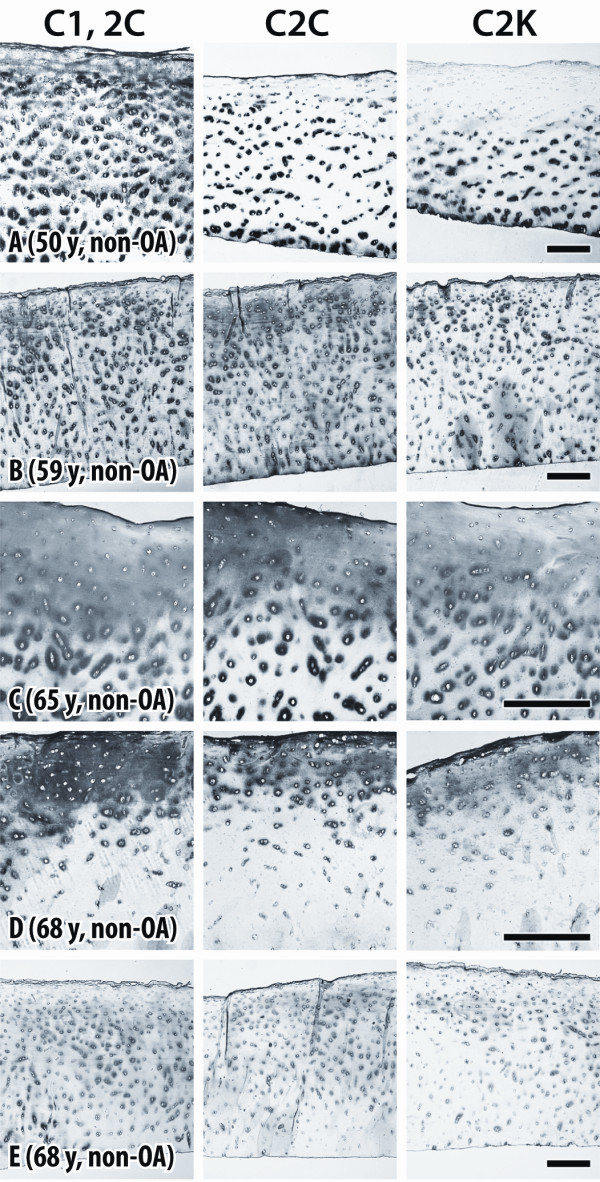
**Immunostaining of cartilage from older normal individuals**. **(A-E) **Sections of human femoral condylar cartilage from five non-OA individuals (ages 50 to 68 years), showing detection of neoepitopes generated by collagenases (C1,2C and C2C) and cathepsin K (C2K) in type II collagen. High-magnification views **(C **and **D) **were used to better indicate pericellular staining. Bars = 200 μm.

Weak to moderate extracellular staining was usually observed in similar sites with all three antibodies around chondrocytes (5/6 cases) in each of the non-arthritic tissues obtained from younger individuals (under 50 years). Staining was seen throughout the full depth of the articular cartilage (Figure 3A, D) or mainly close to the articular surface (Figure 3C, E, F), which was essentially intact. With increasing age (over 49 years), usually more intense pericellular and diffuse extracellular staining was observed, extending from the articular surface into the mid (Figure 4D) and deep zones (Figure 4B). It was more pronounced in pericellular sites but sometimes extended to territorial and interterritorial sites.

The extracellular staining pattern was in general very similar for each of the three antibodies but in most cases a stronger or more diffuse staining was observed for the sections treated with C1,2C antibody compared to C2C, which may result from the recognition of secondary cleavage sites in Col II. Only in one case was cleavage of Col II by cathepsin K different from that detected for collagenases (Figure 4A). Here cleavage of Col II by collagenases was present in the territorial matrix around chondrocytes of all cartilage zones, whereas cleavage by cathepsin K was only located in the mid and deep zones. In this case the cartilage samples varied somewhat between sections due to the difficulty in obtaining acceptable sections. One example (Figure 3B) showed strong pericellular staining for cleavage by collagenases in the superficial and mid zones, as opposed to a thin band of diffuse staining confined to the superficial zone indicating cleavage by cathepsin K.

**Figure 5:**
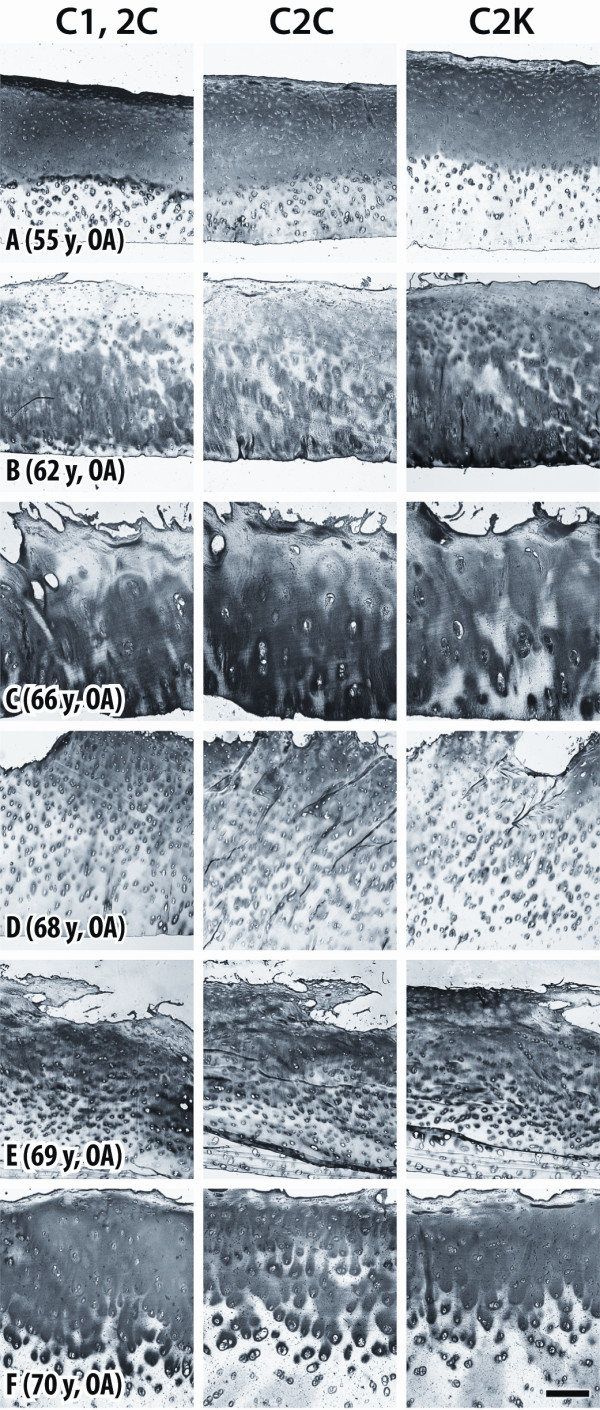
**Immunostaining of cartilage from osteoarthritic patients**. Sections of human femoral condylar cartilage from six OA patients (ages 55 to 70 years), showing detection of neoepitopes generated by collagenases (C1,2C and C2C) and cathepsin K (C2K) in type II collagen. Bar = 200 μm.

OA cartilages exhibited more abundant and widespread intense extracellular staining compared to the non-arthritic group. The staining patterns were different from one case to another but, again, usually similar for all antibodies and in some instances the distribution of staining was similar to, but more pronounced than, that observed in nonarthritic cartilages from older subjects (Figure 5D, F). Pericellular staining extended from the articular surface into the mid and deep zones (Figure 5B, C, E) and extensive diffuse staining present in pericellular, territorial and interterritorial sites was sometimes pronounced in the superficial, mid and upper deep zones (Figure 5A, F).

In other cases (Figure 5B, C), a reduction or loss of staining was observed under the articular surface accompanied by stronger pericellular, territorial and interterritorial staining in the mid and deep zones. Stronger, diffuse staining throughout most of cartilage thickness was observed in both fibrillated cartilages (Figure 5C, E) and in cartilages with intact articular surface (Figure 5A). Figure 5A shows a very clear delimitation between the strong, diffuse and extensive matrix staining in the zone of enhanced collagen cleavage compared to staining deeper in the cartilage where it is only pericellular.

## Discussion

Osteoarthritis with its accompanying cartilage degeneration is a common consequence of aging [20]. Many components of the tissue matrix undergo aging changes, including the major proteoglycan, aggrecan [21], the various collagenous components [22] and the chondrocytes themselves [23]. Excessive proteolysis has long been recognized as a major contributory factor to cartilage matrix degradation in OA. A variety of proteases have been implicated, ranging from lysosomal cysteine proteases, such as cathepsin B, which can cleave aggrecan and non-helical telopeptide sites in Col II, to MMPs, such as MMP-3 (stromelysin), aggrecanases and collagenases [24]. Collagen degradation is considered to be a key process in articular cartilage degeneration in OA [2]. Collagenases are involved in normal extracellular turnover of collagen but their up-regulation is associated with the generation of inflammatory mediators [25], which are believed to play a central role in the pathology of OA [26]. Enhanced Col II degradation by collagenases has been detected in OA articular cartilages [2,27] together with increased denaturation of Col II that results from intrahelical cleavage of Col II [17,18]. It has long been considered that MMPs with the capacity to generate the classic collagenase cleavage site are the principal proteases capable of cleaving triple helical collagen. Recent studies have provided evidence that collagen degradation in health and disease may also involve the action of cathepsin K [4,5,7], a cysteine protease that is up-regulated in OA chondrocytes [3] and is capable of degrading native triple helical collagens, including Col II [4,8], as well as other matrix molecules of the hyaline cartilage, such as aggrecan [28].

The ability of cathepsin K to cleave native Col II at sites in the triple helix distinct from that of the primary collagenase cleavage site [4,8], renders it unique among other tissue proteases. The present study, which used immunoperoxidase localization to identify and compare the distribution of Col II cleavage neoepitopes generated by these different proteases in healthy and OA cartilages, was prefaced by analytical immunoassays of these articular cartilages with the antibodies C2C and C2K, which revealed a significant increase in the generation of these two neoepitopes in aging and more so in OA cartilage [4]. Using immunolocalization in the present study, we were able to demonstrate the more widespread distribution of Col II cleavage in arthritic cartilage and to identify the sites of more intense proteolytic activity within the tissue. Moreover, we showed that sites of cleavage of Col II by collagenases are usually co-localized with sites where the cathepsin K cleavage neoepitope is also found, suggesting that different proteases are active in sites of Col II cleavage in both normal and arthritic cartilages.

Our results for the collagenase neoepitope analyses complement our earlier data [18] and demonstrate that in the majority of cases similar results were obtained with both C1,2C and C2C antibodies. In the cases where differences were observed with more staining for C1,2C, this may result from secondary cleavages in the collagen α chain (Figure 1), which would only be recognized by the C1,2C antibody. Our results further indicate that Col II degradation usually starts around chondrocytes, in the upper region of cartilage, at and close to the articular surface, with progressive involvement of the mid and deep zones in aging and OA. A "stepwise" activation of chondrocytes by cytokines initiated in the superficial cartilage, slowly extending through chondrocyte activation into the deeper zones may account for the clear demarcation between the severely degenerate extracellular matrix and the more normal underlying cartilage. This "creeping substitution" of good with bad appears to be the manner whereby cartilage damage extends ever deeper into the cartilage with time. However, which cytokines stimulate cathepsin K expression in chondrocytes remains to be determined.

In most specimens that were used for the study, a similar distribution was observed for both collagenase and cathepsin K-generated cleavage of Col II. The sites of collagen degradation by collagenases are known to correlate with the distribution of the collagenases MMP-1 and MMP-13 in both healthy and OA cartilages [18]. On the other hand, cathepsin K positive cells have been identified in all zones of degraded cartilage and there is evidence that cathepsin K may be released into the extracellular matrix, as indicated by immunohistochemical detections of the enzyme in the territorial matrix around cells [3]. Recently, in a study of equine OA, use of the same antiserum to the neoepitope generated by cathepsin K revealed that sites of extracellular collagen cleavage were increased in degenerate cartilage and co-localized with cathepsin K staining [29]. These and our present and recent findings [4] together indicate that cleavage of Col II as detected in this study can involve both collagenases and cathepsin K that originate from chondrocytes.

Concomitant with aging in human cartilage, the accumulation of advanced glycation end products has been shown to decrease the susceptibility of this tissue to proteolytic degradation [30,31]. Our immunohistochemical findings are consistent with this progressive crosslinking of the cartilage matrix, which limits the ability of cleavage products to be released from the tissue. This appears to account for the increased staining intensity of the collagen neoepitopes with age and the requirement for extensive chymotrypsin pretreatment of the tissue for immunoassay quantitation. In addition, despite a major effort, we have been unable to biochemically characterize the collagen peptides bearing the cathepsin K neoepitope. The product of *in vitro *collagen cleavage was detectable by immunoassay but was not demonstrable by Western blotting even when high percentage polyacrylamide gels were used.

The present study mainly provides information regarding the distribution of collagen degradation within the tissue. Making quantitative estimates to compare the amount of epitopes generated by each of the enzymes is not possible since the antibody dilutions were adjusted to give the strongest specific antigen staining with the lowest non-specific background. However, our recent quantitative analyses also revealed a significant correlation between cleavage generated by collagenases and that which can be generated by cathepsin K [4]. The present study indicates that Col II cleaving activity of the kind generated by cathepsin K is usually evident in the same sites as collagenase activity in both normal and diseased cartilages. Both studies thus revealed that neoepitopes are generated by both collagenases and cathepsin K in the same sites and increase with age and more so with OA and that these events are correlated in some way. That this non-collagenase cleavage is produced by cathepsin K is supported by our recent observation that the generation of the cathepsin K neoepitope in OA cartilage from some patients in culture can be inhibited by a specific non-toxic inactivator of this cysteine protease [4]. In patients where inhibition was not observed, it appears that cathepsin K was not active in that period of time. This does not preclude the enzyme from being active at other times in these patients. But it also raises the issue of whether proteases other than cathepsin K and collagenases, the latter having not been shown by us to generate this cathepsin K cleavage neoepitope, may also produce this neoepitope. In fact, there have been several studies which have identified other cleavage sites in Col II distinct from those described here with no evidence for a single protease being involved in their generation [32-34]. In a recent proteomic study [35], products of human articular cartilage digested *in vitro *with a series of MMPs and aggrecanases were characterized. While a large number of Col II cleavage products were identified, none were compatible with cathepsin K activity. Thus, only the collagenase generated neoepitope would appear to be specific for this protease [2,14].

Together these results suggest that whereas collagenases may be the dominant mediator of Col II, cleavage in a majority of patients, cathepsin K and other proteases may also play a significant role. In both normal aging and in cartilage degeneration accompanying osteoarthritis, the degradative processes mediating collagen cleavage by these enzymes appear to be similar.

## Conclusions

Collagen degradation is a hallmark of cartilage destruction in osteoarthritis. In addition to the well-accepted role of matrix metalloproteinase collagenases in this process, immunohistochemical staining of cartilage from osteoarthritis patients, together with previous immunoassay data demonstrates the role for cathepsin K in this process. The similarity in distribution of the cleavage products attributed to the two different enzyme types suggests their production by the same cells at and close to the articular surface and that the destruction of collagen is mediated by similar mechanisms during normal aging and osteoarthritis.

## Abbreviations

BSA: bovine serum albumin; C2C and C1,2C: epitopes generated at the new C-terminus of type II collagen and types I and II collagen respectively following cleavage by collagenase in the triple helical region (see Figure 1); C2K: epitope generated at a new C-terminal fragment of type II collagen following cleavage by cathepsin K; Col II: type II collagen; MMP: matrix metalloproteinase; OA: osteoarthritis; PBS: phosphate buffered saline; TC^A^: 3/4 cleavage fragment generated on cleavage of the collagen molecule by the action of collagenases

## Competing interests

A patent application has been filed for the use of the C2K antibody for immunoassay of this epitope in body fluids.

## Authors' contributions

VMD, JSM, SL and ARP designed the study and analyzed the data. VMD carried out the immunohistochemical studies. VMD, JSM and ARP drafted the manuscript. JA, DJZ and MT provided surgical specimens and clinical information. All authors have read and approved the manuscript.
